# Supplementary material for: An insect-like mushroom body in a crustacean brain
Source: eLife. 2017 Sep 26;6:e29889. doi: 10.7554/eLife.29889 (PMC5614564; doi:10.7554/eLife.29889)
Supplement: Transparent reporting form [file elife-29889-transrepform.docx]

***eLife’s* transparent reporting form**

We encourage authors to provide detailed information *within their submission* to facilitate the interpretation and replication of experiments. If you have any questions, please contact us: [editorial@elifesciences.org](mailto:editorial@elifesciences.org).

**Sample-size estimation**

- You should state whether an appropriate sample size was computed when the study was being designed
- You should state the statistical method of sample size computation and any required assumptions
- If no explicit power analysis was used, you should describe how you decided what sample (replicate) size (number) to use

Please outline where this information can be found within the submission (e.g., page numbers or figure legends), or explain why this information doesn’t apply to your submission:

Sample sizes were determined from prior experience with Golgi impregnations, a stochastic method requiring many specimens.

Number of specimens and number of treatments are given in the Methods section, lines 890-908.

Cell body densities counted within a defined volume: populations were calculated from these to give cell packing densities within one cubic millimeter. The relevant description is given in the Method section, lines 991-1006.

Reconstructions of the intact mushroom body were achieved from 220 stacked 12 µm-thick serial silver-stained and interpolated profiles processed using ImageJ. Sections were aligned using multiple fiducial landmarks of non-neural structures (blood vessels). Selected regions were segmented using the TrakEM2 plugin in Fiji (*90,91*) using the TrakEM2 measuring tool to calculate the total volume. These are described in the Methods section lines 986-990 and 998-1006.

The fossil brain of F. protensa was derived from the overlay of images from two specimens YKLP 11357 and YKLP 15006 from the Yunnan Key Laboratory of Palaeobiology, Kunming, China (see lines Method 1007-1011). See Figure 5 supplement 3.

**Replicates**

- You should report how often each experiment was performed
- You should include a definition of biological versus technical replication
- The data obtained should be provided and sufficient information should be provided to indicate the number of independent biological and/or technical replicates
- If you encountered any outliers, you should describe how these were handled
- Criteria for exclusion/inclusion of data should be clearly stated
- High-throughput sequence data should be uploaded before submission, with a private link for reviewers provided (these are available from both GEO and ArrayExpress)

Please outline where this information can be found within the submission (e.g., page numbers or figure legends), or explain why this information doesn’t apply to your submission:

The volume of neurohistological and immunocytological specimens generated is provided in the Methods section, lines 1044-1062.

No specimens were rejected from evaluated data.

**Statistical reporting**

- Statistical analysis methods should be described and justified
- Raw data should be presented in figures whenever informative to do so (typically when N per group is less than 10)
- For each experiment, you should identify the statistical tests used, exact values of N, definitions of center, methods of multiple test correction, and dispersion and precision measures (e.g., mean, median, SD, SEM, confidence intervals; and, for the major substantive results, a measure of effect size (e.g., Pearson's r, Cohen's d)
- Report exact p-values wherever possible alongside the summary statistics and 95% confidence intervals. These should be reported for all key questions and not only when the p-value is less than 0.05.

Calculations of absolute number of neuronal cell bodies relating to mushroom bodies of insect and stomatopod provided in the Method section, lines 1168-1183 are used as Character 1 in the character list, Table 1, main narrative.

Please outline where this information can be found within the submission (e.g., page numbers or figure legends), or explain why this information doesn’t apply to your submission:

(For large datasets, or papers with a very large number of statistical tests, you may upload a single table file with tests, Ns, etc., with reference to page numbers in the manuscript.)

**Additional data files (“source data”)**

- We encourage you to upload relevant additional data files, such as numerical data that are represented as a graph in a figure, or as a summary table
- Where provided, these should be in the most useful format, and they can be uploaded as “Source data” files linked to a main figure or table
- Include model definition files including the full list of parameters used
- Include code used for data analysis (e.g., R, MatLab)
- Avoid stating that data files are “available upon request”

Please indicate the figures or tables for which source data files have been provided:

n/a
